# Distribution of Hyperpolarized Xenon in the Brain Following Sensory Stimulation: Preliminary MRI Findings

**DOI:** 10.1371/journal.pone.0021607

**Published:** 2011-07-15

**Authors:** Mary L. Mazzanti, Ronn P. Walvick, Xin Zhou, Yanping Sun, Niral Shah, Joey Mansour, Jessica Gereige, Mitchell S. Albert

**Affiliations:** 1 Department of Radiology, Brigham and Women's Hospital, Harvard Medical School, Boston, Massachusetts, United States of America; 2 Wuhan Center for Magnetic Resonance, State Key Laboratory of Magnetic Resonance and Atomic and Molecular Physics, Wuhan Institute of Physics and Mathematics, Chinese Academy of Sciences, Wuhan, China; 3 Lurie Family Imaging Center, Center for Biomedical Imaging in Oncology, Dana Farber Cancer Institute, Massachusetts, United States of America; 4 Thunder Bay Regional Research Institute, Thunder Bay, Ontario, Canada; National Institute of Health, United States of America

## Abstract

In hyperpolarized xenon magnetic resonance imaging (HP ^129^Xe MRI), the inhaled spin-1/2 isotope of xenon gas is used to generate the MR signal. Because hyperpolarized xenon is an MR signal source with properties very different from those generated from water-protons, HP ^129^Xe MRI may yield structural and functional information not detectable by conventional proton-based MRI methods. Here we demonstrate the differential distribution of HP ^129^Xe in the cerebral cortex of the rat following a pain stimulus evoked in the animal's forepaw. Areas of higher HP ^129^Xe signal corresponded to those areas previously demonstrated by conventional functional MRI (fMRI) methods as being activated by a forepaw pain stimulus. The percent increase in HP ^129^Xe signal over baseline was 13–28%, and was detectable with a single set of pre and post stimulus images. Recent innovations in the production of highly polarized ^129^Xe should make feasible the emergence of HP ^129^Xe MRI as a viable adjunct method to conventional MRI for the study of brain function and disease.

## Introduction

Although not inherent to biological tissue, the spin ½ nucleus of the isotope of xenon (^129^Xe) is made detectable by magnetic resonance spectroscopy (MRS) and MRI in animals and humans by prior ex-*vivo* hyperpolarization of ^129^Xe through spin-exchange optical pumping which increases its magnetization by up to five orders of magnitude [1], [2]. Although the resulting *in vivo* signal to noise ratio (SNR) of the HP ^129^Xe signal is not as great as the signal produced by protons in conventional MRI, HP ^129^Xe has several unique characteristics which may endow it with advantages in some imaging applications [3], including brain imaging [4]. The nuclear magnetic resonance frequency range (chemical shift) of HP ^129^Xe *in vivo* is large compared to protons (200 ppm vs. 5 ppm respectively) and is also substantially affected by the local chemical environment, providing a means to detect localized physiological changes and biochemical binding events [3]–[5]. In particular, the chemical shift experienced by ^129^Xe in the presence of oxygen (O_2_) is substantial [6], [7] and may offer a means to image changes in tissue O_2_ concentration that result from changes in neuronal activity. Xenon is also an ideal perfusion tracer [8] and inhaled non-radioactive xenon gas has been used to detect disease induced alterations in cerebral blood flow with high anatomical specificity [9]. Because xenon is not intrinsic to biological tissue, HP ^129^Xe produces virtually no background signal, which, in turn, results in high contrast HP ^129^Xe MR images [10]. Lastly, HP ^129^Xe MRI may be beneficial for imaging patients with brain disease or trauma as evidenced by recent findings showing xenon exerts neuroprotective effects against neurotoxic and ischemic damage [11].

Despite these advantages, imaging HP ^129^Xe still faces considerable obstacles owing to the reduction of ^129^Xe T_1_ in the presence of paramagnetic species such as oxygen and blood, and the competing timescale presented by vascular delivery, both of which occur on the order of 10 to 20 seconds [12]. In addition, the obtainable SNR of the ^129^Xe signal in tissues is ultimately limited by the initial level of hyperpolarization obtained *ex vivo* by spin-exchange optical pumping. While improvements in spin-exchange optical pumping techniques [13] and HP ^129^Xe bio-carriers [14], [15] promises to overcome these limitations, the demonstrated usefulness of HP ^129^Xe imaging has remained lacking. Here we show preliminary results mapping changes in the distribution of HP ^129^Xe in the brain following a well-defined fMRI paradigm. Although spatial and temporal resolution was coarse in these preliminary studies, brain areas showing significantly increased HP ^129^Xe signal after a pain stimulus were delineated and found to be the same as those observed using conventional fMRI methods. These results demonstrate that despite low SNR and other limitations, HP ^129^Xe MRI may be useful in detecting physiologically relevant information in the brain.

## Methods

### Hyperpolarized ^129^Xe Generation System

In spin exchange optical pumping, the element rubidium (Rb) is used to transfer the angular momentum from laser light to the noble gas nuclei of ^129^Xe (Happer et al). In this way, large non-equilibrium nuclear spin polarizations can be created. A commercially built hyperpolarized ^129^Xe gas flow-through system (IGI.XE.2000, Amersham Health, Durham, NC) was employed in these studies. Prior to optical pumping, the pumping cell was evacuated to 10^−8^ torr by means of a vacuum-pump system so as to prevent residual oxygen from rapidly depolarizing the HP ^129^Xe gas. An initial gas mixture of natural abundance 1% xenon, 10% nitrogen, and 89% helium was introduced into the glass optical cell via a manifold system. After flowing past a zirconium getter for purification, the gas stream entered the pumping chamber through a Chemglass needle-valve stopcock where polarization occurred at ∼5 atm. The light from a 60 watt diode laser (Coherent Inc. Santa Clara, CA) was circularly polarized using a quarter wave plate and directed into the optical pumping chamber permeated by a magnetic field (20–30 G). Once polarized, approximately one liter of HP ^129^Xe was cryogenically extracted into a holding cell at 77°K, then expanded into a Tedlar bag (Jenson Inert Products, Coral Springs, FL) which was immediately attached to a home-built programmable xenon gas animal delivery system designed for minimal loss of polarization [16]. Polarization of the gas was tested on a calibration system and was routinely found to be between 8% and 11%.

### Delivery of HP ^129^Xe to target tissue

The Harvard Medical Area Standing Committee on Animals has approved all animal procedures (IACUC protocol 03491). Male Sprague-Dawley rats weighing between 200–250 g were initially anaesthetized with an i.p. injection of a ketamine (24 mg/kg) and xylazine (6 mg/kg), and a tracheostomy was performed whereby the airway was catheterized with a 14-gauge, 35 mm catheter. During surgery, the animal's body temperature was maintained at 37°C using a heating pad. The animal was then placed on an animal respirator (SAR 830 AP, CWE Inc., Ardmore, PA, controlled via computer software (LabView, National Inst.) and ventilated with 97% O_2_ at 40 breaths per min with a 400 ms inspiration period, a 250 ms breath-hold period, an 850 ms expiration period, and an inter-breath interval of 1.5 s. A tidal volume of 3 ml was supplied for each breath. 3% isoflurane was added to the O_2_ prior to its delivery to the animal in order to maintain anaesthesia throughout the imaging procedure. During imaging, the animal's body temperature was recorded with a rectal probe (SA Instruments, Stony Brook, NY) and maintained at 37.5±0.5°C with an MRI compatible heating pad (T/Pad and T/Pump, Gaymar Institute, Kent Scientific, Litchfield, CT, USA).

Immediately prior to the acquisition of CSI images, the animal was ventilated with alternate breaths of 100% HP ^129^Xe and 98% O_2_: 2% isoflurane. The breath-hold period during the delivery of each HP ^129^Xe breath was 2 seconds. All gases including HP ^129^Xe were delivered to the animal through a home-built delivery system.

### Image acquisition

**Figure 1:**
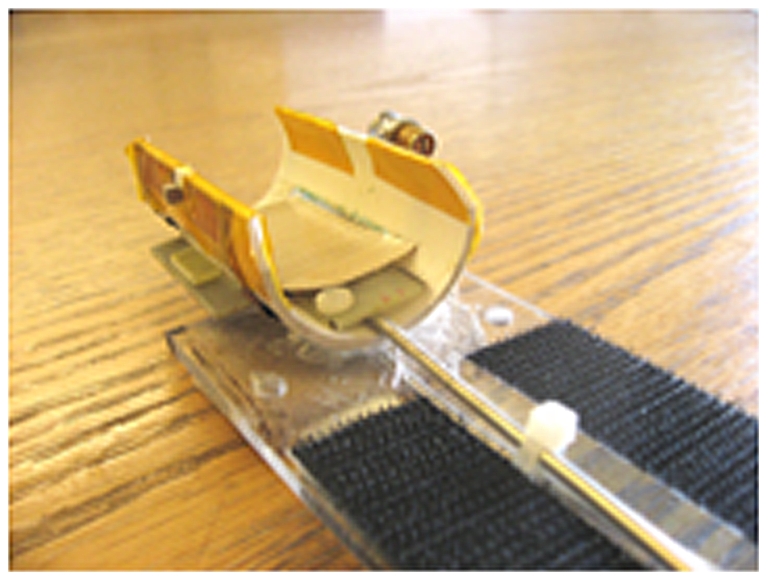
RF coil used for image acquisition. The dual frequency coil combines a Helmholtz pair proton coil (transmit and receive, 200 MHz) nested on a single loop ^129^Xe coil (transmit and receive, 55.35 MHz). This coil architecture allowed proton and ^129^Xe images to be acquired sequentially while maintaining exact co-registration (Clinical MR Solutions, Brookfield, WI). The two coils were intrinsically decoupled.

Imaging was performed on a 4.7 T/33 cm bore Bruker Biospec Advance MRI system controlled by a console running ParaVision software. A dual frequency coil (Figure 1) was used which combined a Helmholtz pair proton coil (transmit and receive, 200 MHz) nested on a single loop Xe coil (transmit and receive, 55.35 MHz) (Clinical MR Solutions, Brookfield, WI). This coil architecture allowed proton and ^129^Xe images to be acquired sequentially while maintaining exact co-registration (Clinical MR Solutions, Brookfield, WI). The two coils were intrinsically decoupled. 1 mm coronal multi-slice proton images through the rat brain were acquired with a fast spin echo sequence (RARE), with TE = 7 ms, TR = 2500 ms, matrix size 128×128, FOV of 25 mm, and 4 averages, during which time (approximately 10 minutes) the animal was ventilated with O_2_: isoflurane. HP ^129^Xe was administered using alternate breaths of ^129^Xe (100%) and the O_2:_isoflurane mixture (98%∶2%). The rise time of the HP ^129^Xe signal in the brain was monitored by the acquisition of the xenon spectral peak evoked by radio-frequency pulses (RF pulse 55.464 kHz, pulse angle 13°, pulse width 85 µs, acquisition points 1024, spectral width 10 kHz, TR 5 sec, no slice selection) and the maximal, steady-state ^129^Xe brain signal occurred within 15 seconds of the start of ventilation with HP ^129^Xe. In one additional animal, an HP ^129^Xe spectrum was acquired with a pulse angle of 90°, and 50 averages. After verification of the xenon signal in the brain, a baseline ^129^Xe chemical shift image (CSI) was acquired that was centred in the plane corresponding to the proton reference image. A 2D CSI sequence was used with 16 and 32 phase encoding steps in the x and y dimensions, respectively, a FOV of 25 mm, a slice thickness of 2.25 or 5 mm, a phase gradient duration of 500 us, a flip angle of 13°, a TR of 500 ms, 256 acquisition points, and one average. Spatial resolution along the x axis was 1.56 mm, and along the y axis was 0.78 mm. Total scan time was 4 min., 16 sec. Because the low flip angle used for CSI acquisition insured minimal loss of HP ^129^Xe signal due to RF destruction, and the relatively long TR allowed continuous delivery of HP ^129^Xe to the tissue, a steady –state concentration of HP ^129^Xe was maintained in the brain thereby insuring constant signal intensity across k-space. K-space data were zero-filled to yield a linear reconstructed image of 128×128 pixels. In a subset of animals (n = 3) the animal's left forepaw was injected with a vehicle solution during baseline. Following acquisition of the baseline CSI image, the animal was ventilated for 10 minutes with O_2_: isoflurane to allow for complete clearance of ^129^Xe magnetization from the brain. Next, the chemical irritant capsaicin (20 ul of 3 mg/ml) was injected into the animal's right forepaw (n = 6), and a second CSI was acquired.

### Image post-processing and statistical analysis

Two dimensional ^129^Xe CSI images with a matrix size of 16×32, were reconstructed using commercial software (ParaVision, Bruker Biospin, Billirica MA), where the magnitude of the signal in each pixel was calculated from the integration of the spectral peak. The goal of CSI is to generate separate images for each spectral peak sampled. Because the 2D CSI sequence used in this study acquired 256 points for each phase encoding step, it resulted in 128 images corresponding to the 128 spectral frequencies sampled. Consistent with previous reports, only the main spectral peak occurring at 194.7 ppm was sufficient in SNR to be used for image generation, and therefore only those images corresponding to that spectral frequency were further processed. Thus resulting images reflect the spatial localization of this primary peak, and show ^129^Xe pixel intensity in units of SNR.

In order to map ^129^Xe pixel intensities to anatomical locations in the brain, the 2D ^129^Xe CSI images were resized using interpolation (Image J, NIH) to match the matrix size of the corresponding proton reference image (128×128) from the same animal. Because both the ^129^Xe and ^1^H signals are taken with the same coil, there was no movement of the anesthetized animal between acquisition of the two images, which allowed exact registration of the two images when reconstructed in this fashion. In order to align ^129^Xe images for computing statistics, the corresponding proton reference images from each animal (n = 6) were manually aligned using a rigid-body transformation without spatial interpolation using QuickVol II software (http://www.quickvol.com), and the alignment parameters used for each ^1^H image were then applied to the ^129^Xe image from the same animal.

To visually assess changes in ^129^Xe signal intensity after the injection of capsaicin in individual animals, anatomical maps of ^129^Xe signal intensity were generated by assigning a colour look up table with 255 discrete brightness values to the original images, and these were presented as colour overlays on the corresponding proton reference image. In order to show the pattern of highest signal changes, only signals over a designated SNR were shown. Additionally, for aesthetic reasons, signal originating outside the boundaries of the surface coil where removed from the final images by using a mask.

The distribution of HP ^129^Xe signal during baseline and after injection of capsaicin was statistically assessed using an analysis of covariance (ANCOVA) [17]. Regions of interest (ROIs) were drawn around the boundaries of anatomically discrete areas of the brain using image processing software (Image J, NIH). The boundaries of the anatomical areas were delineated by tracing the boarders of a standard rat sterotaxic atlas [18] which was overlayed on the HP ^129^Xe image. ROIs were chosen on the basis of previously reported findings which show that specific brain regions are consistently activated after the application of a pain stimulus to the rat forepaw [19]–[24] and these areas were designated as ‘pain-areas’. An equal number of ROIs were chosen in other areas of the brain (lateral and medial septum, piriform cortex, and insular cortex) and designated as ‘non-pain areas’. Measurements from ROIs were normalized by dividing the raw ^129^Xe pixel intensity value by the RMS of the random noise value measured outside the brain for each image, yielding an HP ^129^Xe SNR value for each ROI [10]. In the statistical analysis, pain areas were submitted as dependent variables while non-pain areas acted as the covariate to account for global physiological changes such as heart rate and blood pressure that may affect the ^129^Xe signal [17]. A comparison of HP ^129^Xe signal in the two baseline conditions (saline versus no saline injection) found no significant differences between the groups and these two groups were combined to form one control group (n = 6) for further analysis.

## Results

**Figure 2:**
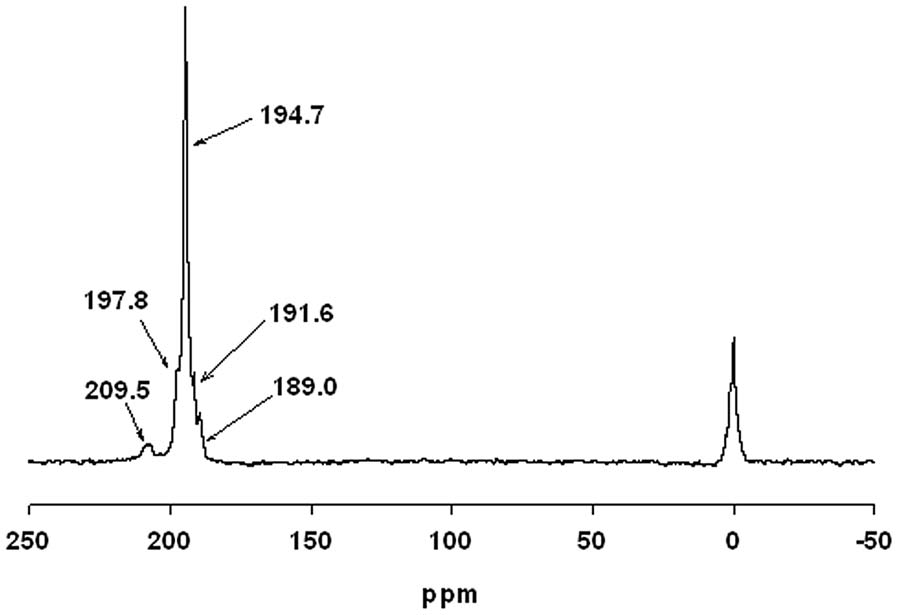
HP ^129^Xe spectrum obtained from rat brain *in vivo* after the administration of HP ^129^Xe gas. The spectrum was acquired from 50 averages using an RF pulse with a flip angle of 90°, and a frequency of 55.477 MHz. At least four separate peaks are discernable, the largest of which occurs at 194.7 ppm downfield from the HP ^129^Xe gas peak at 0 ppm. The SNR of the largest peak is 476.

In this study HP^129^Xe MRI was performed in rats to investigate the distribution of the HP^129^Xe signal following a well-established paradigm for producing anatomically localized neuronal activity. Six rats were intubated and connected to a ventilator that controlled the delivery of oxygen and HP ^129^Xe gas. High-resolution proton images were taken of the rat head to provide an anatomical reference for HP ^129^Xe images. A robust HP ^129^Xe spectroscopic signal (average SNR of 13.21±2.92) with one primary peak at 194.7 ppm developed within 15 seconds of the start of ventilation with HP ^129^Xe. In order to more easily visualize smaller HP ^129^Xe spectroscopic peaks, a spectrum was acquired with 50 averages, resulting in a SNR of 476 for the primary peak at 194.7 ppm, and revealing four additional peaks at 209.5, 197.8, 191.6, and 189.0 ppm (Figure 2). The T_2_* of the primary peak was 5.42±0.3 ms at 4.7T.

**Figure 3:**
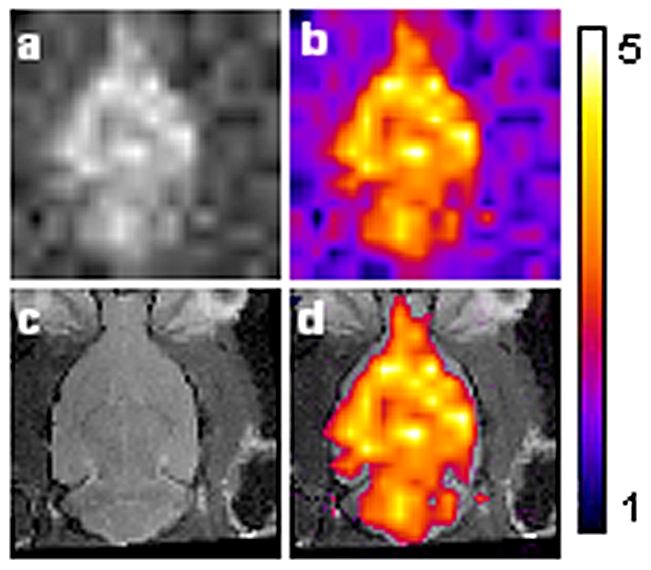
HP ^129^Xe distribution in the rat brain. (**3a**) HP ^129^Xe CSI image acquired with a 2D CSI pulse sequence from rat head under normal breathing conditions (slice thickness 10 mm). (**3b**) same image with false color applied. Warmer colors indicate increased HP ^129^Xe signal intensity. (**3c**) Proton MRI of a rat head showing a 1 mm coronal slice through the brain acquired with a RARE pulse sequence. (**3d**) Proton image shown with overlay of HP ^129^Xe MRI, in which only HP ^129^Xe signal with an SNR above 2 are shown. FOV was 25 mm.

In order to determine the extent of HP ^129^Xe distribution throughout the rat brain, a magnetic resonance spectroscopic (MRS) image was acquired of the primary peak during administration of HP ^129^Xe (Figure 3). The four smaller resonances did not have sufficient SNR to produce spectroscopic images. Figure 3a shows an HP ^129^Xe image taken in the axial plane. Addition of a colour look-up table (Figure 3b) aided in visually delineating areas of low and high SNR. Figure 3c show a 1 mm proton slice in which the olfactory bulbs and cerebellum are visible. Overlay of the HP ^129^Xe spectroscopic image onto the proton reference image (Figure 3d) revealed that the steady-state HP ^129^Xe signal originated from within the brain tissue and further demonstrated a pattern of HP ^129^Xe distribution throughout the brain with varying signal intensity in different brain regions.

**Figure 4:**
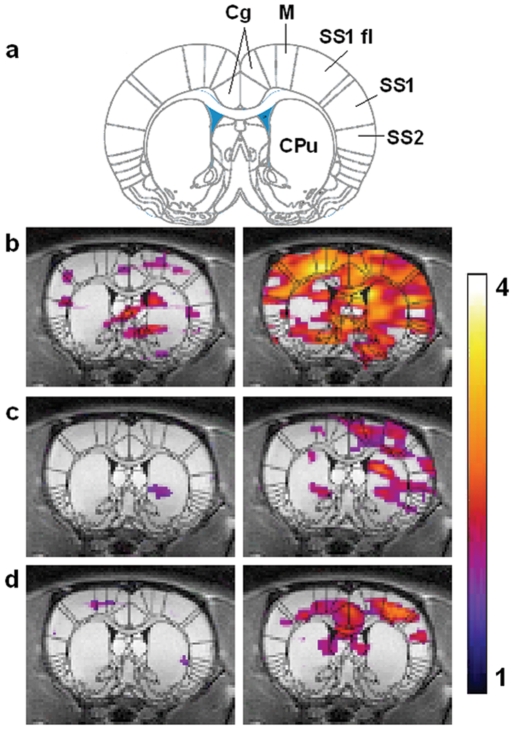
HP ^129^Xe fMRI data from three animals. The HP ^129^Xe signal is shown as a false colour overlay on the corresponding 1 mm thick coronal proton reference image taken from the same animal. The left panel shows HP ^129^Xe signal intensity during baseline and the right panel shows HP ^129^Xe signal intensity after injection of capsaicin 20 ul (3 mg/ml) into the right forepaw. Colour scale represents SNR and only signal with SNR above 2 are shown. Superimposition of a rat brain atlas (18) demarcates specific areas of the brain: cingulate cortex (Cg), motor cortex (M), primary somatosensory cortex and SS1 forelimb region (SS1 and SS1 fl), secondary samatosensory cortex (SS2), and striatum (CPu).

In order to evaluate the distribution of HP ^129^Xe in brain following an external sensory stimulus, we acquired MRS images before and after a pain producing stimulus that has a well-defined functional response that can be measured using traditional fMRI techniques. A baseline HP ^129^Xe spectroscopic image was acquired from a coronal slice centered at the level of the anatomical reference slice (Figure 4, left panel). Three of the six animals studied received a vehicle injection (saline) to the left forepaw immediately prior to the acquisition of the baseline image. 10 minutes after acquisition of the baseline HP ^129^Xe MRS, the animal's right forepaw was injected with the chemical irritant capsaicin (20 µl of 3 mg/ml), and a second HP ^129^Xe spectroscopic image was acquired.

**Figure 5:**
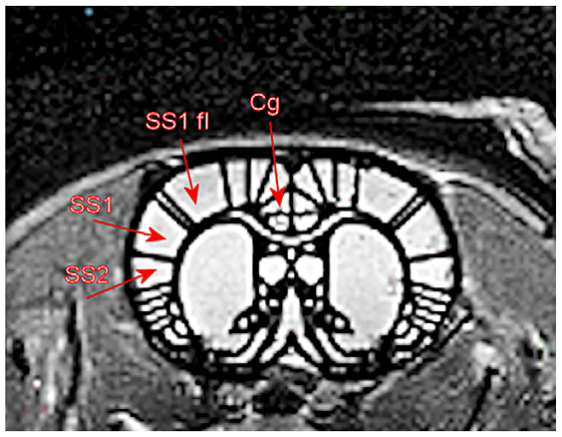
Superimposition of a rat brain atlas [**18**] showing four regions of interest (ROIs) analyzed for changes in HP ^129^Xe signal following forepaw stimulation, including cingulate cortex (Cg), primary somatosensory cortex and SS1 forelimb region (SS1 and SS1 fl), and secondary samatosensory cortex (SS2).

Responses from three individual animals are shown in Figure 4. Whereas baseline images showed some HP ^129^Xe signal intensity in cortical and sub-cortical brain regions (Figure 4, left panel), images acquired following administration of capsaicin showed both higher HP ^129^Xe signal intensity and an increased area of distribution within the brain (Figure 4, right panel). Superimposition of a rat brain atlas (Figure 4a, [18] revealed that areas of HP ^129^Xe signal increase occurred both bilaterally and contralaterally in areas of the brain known to be involved in the processing of forepaw pain information, including the anterior cingulate and somatosensory cortices. Increases for four discrete brain regions are shown in Table 1. Increases were also seen in subcortical regions such as the striatum. ANCOVA analysis of discrete “pain area” ROI's (Figure 5) for six animals resulted in a statistically significant increase for HP ^129^Xe signal in the contralateral anterior cingulate cortex (28.1%±13.5%, p = 0.045). An increase in HP ^129^Xe signal was also seen in the contralateral primary sematosensory cortex (22.16±10.28%, p = 0.055) and the contralateral secondary sematosensory cortex (13.16%±19.36%, p = 0.7), although the later did not reach our *a priori* level of significance. In order to determine if the capsaicin caused changes in heart rate or blood oxygen saturation, these variables were measured at five time points during the acquisition of CSI data in three animals (Table 2). A slight elevation in heart rate (HR) was observed after the injection of capsaicin (439.33±113.43 vs. a baseline value of 356.46±92.04) and was significant (repeated measures ANOVA, p<0.5). There were no significant changes in blood oxygen saturation after the administration of capsaicin.

**Table 1:** Percent HP ^129^Xe signal change in four brain regions*.

	Cingulate	SS1 fl	SS1	SS2
**A1**	76.72	6.19	16.45	12.33
**A2**	40.70	42.69	27.65	5.05
**A3**	31.37	112.59	67.93	−14.07
**A4**	−26.07	−15.06	0.44	106.17
**A5**	23.23	−9.31	0.32	−24.77
**A6**	22.82	24.87	21.71	4.32

*Measured from regions of interest (ROIs) in hemisphere contralateral to pain stimulus.

**Table 2:** Vital Signs Data (N = 3).

Baseline						Capsaicin				
TP1		TP2	TP3	TP4	TP5	TP6	TP7	TP8	TP9	TP10
Heart Rate (beats/min)										
A1	336	344	344	345	346	332	435	443	432	432
A2	379	377	374	375	374	420	414	430	433	440
A3	356	353	355	333	356	449	497	497	483	453
Oxygen Saturation (%)										
A1	98	100	98	99	99	98	97	99	100	100
A2	92	92	92	92	92	94	94	93	94	93
A3	99	98	99	99	99	99	99	99	99	99

## Discussion

In this study we observed the distribution of HP ^129^Xe in the rat brain following a well characterized paradigm for evoking anatomically localized activity in the rat brain [19]–[24] to test the capability of HP ^129^Xe MRI to map these changes. The hypothesis that HP ^129^Xe distribution in the brain may follow a pattern similar to brain activity is based on the well established role of HP ^129^Xe as a perfusion tracer [8], and on the established link (albeit non-linear) between brain activity and blood flow [24]–[26].

Our results show that the HP ^129^Xe signal was increased in many areas of the brain following a pain stimulus and that these areas coincide with those previously found to be activated using conventional BOLD and perfusion based MRI methods. Increases in HP ^129^Xe signal were observed in the primary somatosensory cortex and cingulated cortex contralateral to the forepaw injected, consistent with the activation pattern seen using conventional proton fMRI [19]. HP ^129^Xe signal was also observed in subcortical regions and is consistent with the findings of Governo, et al., [27]. These results suggest that HP ^129^Xe MRI is an imaging modality that may be useful for obtaining physiologically relevant information from the brain, and moreover, that HP ^129^Xe MRI could be developed to provide an alternative means of measuring brain activity with MRI.

Direct comparison of HP ^129^Xe MRI to conventional methods using BOLD and measures of perfusion such as ASL are not meaningful at this time because of the substantial differences between the robustness of the two techniques, and because the underlying mechanisms which determine the distribution of HP ^129^Xe in brain are not fully understood at this time . Because the SNR obtainable with conventional ^1^H MRI is roughly 20 times higher than that obtainable with HP ^129^Xe in this study, the temporal and spatial resolutions obtainable with conventional ^1^H fMRI are not yet obtainable with HP ^129^Xe. Here we obtained a spatial and temporal resolution of 1.56×0.78×5 = 6.08 mm^3^ and 4 min 16 sec respectively, whereas ^1^H fMRI has been used to measure brain activity evoked with this paradigm with a spatial resolution of 0.125 mm^3^ and a temporal resolution of 50s [28].

The somewhat variable response seen from each animal in our preliminary results may represent a confluence of many factors. The stimulus used to induce a pain response is subject to administration variability. More traditional and controllable pain stimuli were inappropriate for this experiment due to the need for a long duration response to accommodate the xenon imaging time. This is potentially complicated by a variable response of each animal to anesthesia which may play a role in modulating the neuro response to the stimulus. Further, xenon polarization has been known to vary from experiment to experiment. Higher polarization will directly lead to higher Xenon SNR in the brain. Avenues for improvement in the HP ^129^Xe SNR *in vivo* include new methods of producing more highly polarized gas [13] and development of biocompatible lipid carriers which can lengthen the T_1_ and T_2_ relaxation time of HP ^129^Xe [15], [29], [30]. Another three fold increase in SNR can be obtained by using xenon which is isotopically enriched to 80–90% ^129^Xe. The impact of increasing levels of ^129^Xe polarization alone should lead to large improvements in signal, as the SNR of the signal in vivo is directly proportional to the level of gas polarization. Whereas the present results were obtained with ^129^Xe polarization levels of about 8 to 11%, polarization of up to 60% is now possible, and thus as much as a five fold increase in SNR obtained in this study is expected to be achievable. Furthermore, three - fold increases in ^129^Xe T_1_ and T_2_ relaxation times have been obtained by dissolving HP ^129^Xe into biocompatible carries [15], [29], [30]. Such increase allow more time for HP ^129^Xe signal to reach the brain and be sampled and as such should also allow for substantial increases in SNR *in vivo* and for the implementation of conventional fast pulse sequences as evidenced by Duhamel and colleagues [14]. An additional factor affecting the HP ^129^Xe SNR *in vivo* is the O_2_ concentration in the breathing gas mixture. While lowering the concentration of O_2_ in the breathing gas mixture will prolong the T1 of ^129^Xe in the gas phase in the lungs, it also decreases the concentration of O_2_ in the blood (transporting the xenon from the lungs to the brain) which paradoxically, actually shortens the T1 in the blood. Thus the concentration of O_2_ in the breathing gas mixture must be chosen carefully. In this study, we chose to use a relatively high O_2_ concentration in the breathing mixture in order to insure the animal maintained a high oxygen saturation value, since ventilating the animal on room air drops the O_2_ sat to unhealthy levels. Pilot studies performed by our group (unpublished) suggest that the overall effect of the O_2_ concentration in the gas versus dissolved phase can be measured empirically, and that such measurements should enable the optimization of the HP ^129^Xe T1 *in vivo*.

In spite of the as yet unrefined nature of this imaging modality, our results indicate that HP ^129^Xe MRI may have use as a probe for brain physiology and function. Because xenon is not inherent in the body, the substantial challenges resulting from high background signal in ^1^H fMRI may be somewhat reduced. Extracting meaningful data from ^1^H fMRI experiments is labour intensive, and requires a large number of subjects and image acquisitions. Extensive image post-processing is required and the influence that different post-processing steps play on the final data set achieved is actively debated. Conversely, HP ^129^Xe MRI showed patterns of brain activation consistent with those obtained using H fMRI, using only a single set of images (one baseline and one post stimulus image) obtained from six animals. The magnitude of the signal difference between baseline and stimulus conditions for HP ^129^Xe (13–28%) was comparable to differences typically obtained with conventional BOLD fMRI (2 to 29%) [20]–[23] using a rat forepaw activation paradigm.

The magnitude of single increases reported here may be a slight underestimate of those ultimately obtainable using HP ^129^Xe fMRI. This is because a slight *decrease* of the signal in the second image is expected due to the occurrence of some T_1_ relaxation of the hyperpolarized xenon in the tedlar bag reservoir during the time interval between the baseline and capsaicin-activated images. However because this time interval is short compared to the T_1_ of HP ^129^Xe in the tedlar bag (10 minutes versus a T_1_ of 1.5 hours), signal loss in the second image due to T_1_ relaxation should be relatively small (∼10%), and thus the signal increases reported here are not likely to be a gross underestimate of the true increase evoked by capsaicin. While the slight increase in heart rate occurring after capsaicin injection may have resulted in increased delivery of HP ^129^Xe to the brain, this increase is presumed to be global, and therefore unlikely to account for anatomically localized increases in HP ^129^Xe distribution in the brain. Furthermore, global changes affecting the HP ^129^Xe signal can be accounted for statistically with the use of ANCOVA [17].

Although xenon's anaesthetic properties could complicate fMRI studies, it is likely that with highly polarized gas [13], [31], and/or the use of isotopically enriched ^129^Xe, imaging will be feasible with concentrations of 40% or less. In contrast, a minimum alveolar concentration of 71% must be reached in humans to induce full anaesthesia [32], [33], whereas lower concentrations (28–35%) used in computerized tomography (CT) produce only slight alterations in sensorium which most patients report as pleasant [34]. It has been shown that functional brain activation evoked during visual stimulation is not significantly altered by the inhalation of 33% Xe [34]. Nevertheless, delineating the effects of even low concentrations of xenon on overall brain metabolism and function will be important for the correct interpretation of HP ^129^Xe fMRI data. A recent study by Laitio et. al. [35] showed that administration of xenon (63%) in humans decreased rCBF in the cerebellum, thalamus, and cortical areas, while increasing rCBF in white matter and in parts of the precentral and postcentral gyri. Based on work by Rex et. al. [36] showing that xenon administration is followed by a global reduction in regional cerebral glucose metabolism, Laitio and colleagues speculated that reductions in rCBF may steam from the reduced activity, thus metabolism, of anesthetized brain cells.

The exact mechanism whereby HP ^129^Xe maps areas of increased neuronal activity is unknown, but likely results from increases in blood flow and blood volume, and/or tissue O_2_ content evoked by neuronal activity. Xenon is an ideal perfusion tracer [8] and HP ^129^Xe has been used to obtain absolute measures of rCBF in rat brain [37] with a spatial and temporal resolution of 1.3 mm and 1 second, respectively. Thus fMRI based solely on measures of rCBF by HP ^129^Xe may prove to be a highly quantitative and accurate method for studying brain functional activation, given that changes in rCBF are highly correlated, both spatial and temporal, to the activity of neurons [24]–[26]. Implementation of this approach in large scale studies should become possible with greater access to highly polarized gas and the design of new biocompatible HP ^129^Xe carrier agents.

The identity of the primary ^129^Xe spectral peak from the brain was not unequivocally identified in this study, but its resonance frequency at 195 ppm is in agreement to previous reports of HP ^129^Xe dissolved in brain tissue [10], [38]. The designation of this peak to grey matter [39] is consistent with the wide-spread distribution of HP ^129^ Xe in the brain (Figure 3) and the predominance of grey matter to white matter in the rat brain. Our results are also in excellent agreement to previous studies which have shown four addition resonance frequencies measured from rat brain at 210, 198, 192 and 189 ppm [10], [38]. The resonances at 210 and 189 ppm are believed to arise from blood and non-brain tissue, respectively.

In addition to enabling novel fMRI studies, HP ^129^Xe might also serve as an adjunct to conventional MRI and MRS in the detection of brain disease. MRI is increasing being used as a primary diagnostic test for stroke. As an ideal perfusion tracer, HP ^129^Xe MRI may be useful for detecting altered cerebral blood flow in neurovascular disease and stroke, without the need for administration of contrast agent which carries the risk, albeit low, of toxicity. Furthermore, because xenon is neuroprotective, HP ^129^Xe MRI might allow diagnostic information to be obtained while saving vulnerable brain tissue in these patients [11]. Other characteristics of HP ^129^Xe may also prove useful in brain imaging such as its differential solubility in white and grey matter (partition coefficient λ, 1.4 vs. 0.6 respectively [28] which may provide a means of generating contrast between these tissues. The relaxation time constants (T_1_ and T_2_) of HP ^129^Xe can be markedly different in different tissues, and thus can be used to generate soft tissue contrast by T_1_ and T_2_ weighting [40].

The recent development of HP ^129^Xe as a specific reporter of biomolecules and ligand-receptor binding [3], [5] further adds to the myriad of possibilities for the use of HP ^129^Xe MRI in disease targeted imaging. Continued development of HP ^129^Xe MRI [41] should render it a valuable adjunct imaging technique capable of revealing additional structural, chemical, and functional information from magnetic resonance studies of the brain.
